# Application of Somatic Embryogenesis in Woody Plants.

**DOI:** 10.3389/fpls.2016.00938

**Published:** 2016-06-24

**Authors:** Yuan Guan, Shui-Gen Li, Xiao-Fen Fan, Zhen-Hong Su

**Affiliations:** Forestry and Fruit Tree Research Institute, Shanghai Academy of Agricultural SciencesShanghai, China

**Keywords:** somatic embryogenesis, embryogenic cell, somatic embryo, woody plants, mass propagation, synthetic seeds, cryopreservation, regulatory genes

## Abstract

Somatic embryogenesis is a developmental process where a plant somatic cell can dedifferentiate to a totipotent embryonic stem cell that has the ability to give rise to an embryo under appropriate conditions. This new embryo can further develop into a whole plant. In woody plants, somatic embryogenesis plays a critical role in clonal propagation and is a powerful tool for synthetic seed production, germplasm conservation, and cryopreservation. A key step in somatic embryogenesis is the transition of cell fate from a somatic cell to embryo cell. Although somatic embryogenesis has already been widely used in a number of woody species, propagating adult woody plants remains difficult. In this review, we focus on molecular mechanisms of somatic embryogenesis and its practical applications in economic woody plants. Furthermore, we propose a strategy to improve the process of somatic embryogenesis using molecular means.

## Introduction of Somatic Embryogenesis in Plants

In flowering plants, the process of double fertilization involves a haploid sperm fertilizing a haploid egg cell to form a diploid zygote. Subsequently, the zygote undergoes a series of morphological, biochemical, and molecular events to develop into an embryo. This stage of development is referred to as embryogenesis (Goldberg et al., 1994). Somatic embryogenesis is when a somatic cell dedifferentiates to a totipotent embryonic stem cell that can give rise to an embryo *in vitro* (Verdeil et al., 2007; Ikeuchi et al., 2015). Since the original description of somatic embryogenesis in carrot (*Daucus carot*) cell cultures (Steward et al., 1958), this process has been reported in various plant species (Gleddie et al., 1983; Tautorus et al., 1991; Jain et al., 2000). The developmental stages of somatic embryogenesis are similar to the process of zygotic embryogenesis in terms of developmental and regulatory mechanisms (Dodeman et al., 1997). Therefore, somatic embryogenesis can provide an accessible model for studying the earliest developmental events of the zygotic embryo in the lifecycle of higher plants (Zimmerman, 1993). Somatic embryogenesis plays a significant role in mass propagation *in vitro*, germplasm conservation, and genetic improvement of woody plants (Merkle and Dean, 2000; Chiancone and Germanà, 2013; Ozudogru and Lambardi, 2016).

Somatic and zygotic embryos have similar developmental stages typically passing through globular, torpedo, and cotyledonary stages in dicots, or globular, scutellar, and coleoptilar stages for monocots (Mordhorst et al., 1997). For conifers, morphogenetic stages include globular, early cotyledonary and late cotyledonary embryos stages (Quiroz-Figueroa et al., 2006). There are two different ways of inducting somatic embryogenesis including direct somatic embryogenesis and indirect somatic embryogenesis (Yang and Zhang, 2010). In direct somatic embryogenesis, somatic embryos can be directly induced from the explant under certain conditions without any intermediate callus stage. Conversely, indirect somatic embryo genesis occurs via an intermediate callus stage and has been observed in most species (Cuenca et al., 1999; Ikeda-Iwai et al., 2002; Gaj, 2004; Montalbán et al., 2012; Corredoira et al., 2013, 2015). Distinguishing between direct and indirect somatic embryogenesis can be difficult as both processes have been observed to occur simultaneously in the same tissue culture conditions (Turgut et al., 1998; Gaj, 2004). In contrast to primary somatic embryogenesis induced from explant cells, secondary somatic embryogenesis is the phenomenon whereby new somatic embryos are induced through existing somatic embryos (Raemakers et al., 1995). In woody plants, secondary somatic embryogenesis can maintain the embryogenic competence of cultures for many years and thus provide useful research material (Martinelli et al., 2001; Martínez et al., 2015). Indirect somatic embryogenesis is a multi-step regeneration process beginning with a proembryogenic mass (PEM), followed by somatic embryo formation, maturation, and conversion (von Arnold et al., 2002). A key point in indirect somatic embryogenesis is the production of PEM consisting of proliferating embryogenic cells at an intermediate state between callus and somatic embryo and a relatively disorganized structure (Halperin, 1966). Auxin is required for the proliferation of PEMs but inhibits the development of PEMs into somatic embryos (Yang and Zhang, 2010).

Given that the potential applications of somatic embryo genesis in woody plants span a broad range of topics, this review will focus on briefly introducing practical applications of somatic embryogenesis in economically significant woody plants. Molecular mechanisms to improve the development of somatic embryogenesis in woody plants will be also discussed.

## Application of Somatic Embryogenesis in Woody Plants

The rapid increase in human population size, environmental pollution, and demand for timber products has put enormous pressure on trees. Development of new technologies for tree propagation, improvement, and breeding can help to solve these problems (Timmis, 1998). This has been achieved in part using biotechnology methodologies like *in vitro* propagation, genetic transformation, and marker-assisted breeding to gradually genetically improve woody plants (Merkle and Dean, 2000; Lelu-Walter et al., 2013). *In vitro* propagation could not only be used for mass clonal propagation of desirable genotypes, but could also provide suitable target material for genetic transformation (Rugh et al., 1998; Vidal et al., 2010).

### Somatic Embryogenesis Is the Preferred Method for *In Vitro* Propagation of Woody Plants

Although shoot proliferation methods, using adventitious shoots and axillary buds have been widely applied to *in vitro* propagation, research is still largely focused on using somatic embryogenesis in woody plants (Germanà and Lambardi, 2016). This technology is important for woody plants that have a long life cycle and are difficult to propagate by conventional methods (Isah, 2016). In tissue cultures, plant regeneration via somatic embryogenesis may offer many advantages over organogenesis, such as the feasibility of single cell origin and the possibility of automating the large-scale production of embryos in bioreactors and field planting as synthetic seeds (Giri et al., 2004). The bipolar nature of embryos allows for direct development into plantlets without the need for the rooting stage required for plant regeneration organogenesis (von Arnold et al., 2002). Furthermore, single epidermal cell origins for embryos might avoid chimeras, favoring the use of this process for plant transformation (Normah et al., 2013).

In woody plants, pioneering research on somatic embryo genesis was only observed to form the embryo-like structures in sandalwood (*Santalum album* L.; Rao, 1965) and several conifers (Durzan and Steward, 1968; Chalupa and Durzan, 1973). However, these embryo-like tissues did not grow into complete plants. Early reports of regeneration via somatic embryogenesis of sandalwood were achieved using hypocotyl and nodal segments (Bapat and Rao, 1979; Sita et al., 1979). In conifers, somatic embryogenesis and plantlet regeneration were first reported in Norway spruce (*Picea acies*), for which immature and mature zygotic embryos were used as explants to establish a culture system (Chalupa, 1985; Hakman et al., 1985). This was followed by extensive studies into exploiting the potential of somatic embryogenesis of important tree species with examples are described below.

The embryogenic tissue of loblolly pine (*Pinus taeda*) was initiated from immature zygotic embryos on media with 3 mg/L 2,4-dichlorophenoxy acetic acid (2,4-D) and 0.5 mg/L 6-benzyladenine (BA). Somatic embryo development was then progressed to the precotyledonary stage on media with 2.6 mg/L abscisic acid (ABA; Becwar et al., 1990). To increase the efficiency of somatic embryogenesis, cold storage has been used to improve the competence of somatic embryogenesis initiation in several *pinus* species (Häggman et al., 1999; Montalbán et al., 2015). Cold stored radiata pine (*pinus radiate*) plant material had increased somatic embryo initiation rates, potentially caused by epigenetic changes in plant tissues that were triggered by temperature stress (Montalbán et al., 2015). Embryogenic calli of Japanese larch (*Larix kaempferi*) were obtained from mature embryos cultured in dark condition on Quoirin and Lepoivre media with 1.0 mg/L 4-amino-3,5,6-trichloropicolinic acid (Picloram) and 1.0 mg/L 6-BA (Kim, 2015). Embryogenic calli of Norway spruce were initiated from immature zygotic embryos on medium containing 10 μM 2,4-D and 5 μM 6-BA (Hakman et al., 1985). Further improvements of somatic embryo development show that the highest plantlets yield was obtained calli were cultured on half strength Quoirin and Lepoivre media containing 90 mM sucrose and 7.6 μM ABA for one month (von Arnold and Hakman, 1988).

Eucalypts are widely planted hardwood forest trees because of their fast growth and remarkable adaptability (Patt et al., 2006). Eucalypts can also provide excellent resources in terms of the production of pulp and eucalyptus oil. In *Eucalyptus* species, most reports were on embryogenic cells derived from immature and mature zygotic embryos or juvenile seedlings (Prakash and Gurumurthi, 2010). Somatic embryogenesis achieved induction using leaf from mature trees is rarely reported. A recent study has described somatic embryogenesis induction from leaf and shoot apex explants of mature *Eucalyptus globulus* and hybrid *E. saligna* × *E. maidenii* plants, with the use of 40 μM Picloram resulting in a higher frequency of *in vitro* culturing than using 2,4-D (Corredoira et al., 2015).

The East Indian sandalwood tree (*S. album* L.) is costly heartwood that is the source of sandalwood essential oil. Somatic embryogenesis provides a system for large-scale plant propagation in bioreactors (da Silva et al., 2016). Misra and Dey (2013) reported an efficient protocol for the mass production of sandalwood biomass by bioreactor based cultivation of somatic embryogenesis. Somatic embryos can be used to produce raw medicinal materials such as santalols, phenolics, and arabinogalactan proteins. A successful rapid protocol for somatic embryogenesis using cultures of nodal segments on media with 2.5 mg/L 2,4-D and 3 mg/L kinetin was recently described using elite trees (Peeris and Senarath, 2015).

The strawberry tree (*Arbutus unedo* L.) is a very important perennial shrub or small tree in different ecosystems and is an attractive ornamental plant. The fruit of the strawberry tree are commonly consumed fresh or processed into jam (El-Mahrouk et al., 2010). Conventional methods of propagation cannot preserve elite strawberry tree genotypes. Somatic embryogenesis is able to overcome this problem by using of leaves from adult trees to induce somatic embryos (Martins et al., 2016). Alders species are of minor importance in economic terms, but do have ecological value through land reclamation and reforestation. Alders can fix atmospheric nitrogen through a symbiotic association with the actinomycete (Oliveira et al., 2005). Corredoira et al. (2013) first reported being able to achieve plant regeneration from immature zygotic embryos through somatic embryogenesis in black alders (*Alnus glutinosa*). Induction media were composed of 0.9 μM 2,4-D and 2.22 μM BA. For medicinal woody plants, such as bastard teak (*Butea monosperma* (Lam.) Kuntze), somatic embryogenesis was able to effectively produce a large number of plantlets and bioactive compounds. Moreover, qualitative analysis using Liquid chromatography electro spray ionization quadrupole time of flight mass spectrometry (LC ESI Q-TOF MS) showed that the secondary metabolites *in vitro* developed cultures were the same as those in wild grown leaf samples (Sharma et al., 2015).

The developmental pattern of somatic embryos is very similar among most woody species tested to date. This begins with an immature or mature embryo that is cultured on a nutrient medium containing a high concentration of plant growth regulators like 2,4-D, 6-BA, and Picloram (Isah, 2016). It is disadvantageous to use immature or mature zygotic embryos as explants because of their unproven genetic value. Although induction of somatic embryo using leaf from mature tree as explants have already been achieved in a small number of species, this difficulty still remains unsolved in initiation of embryogenic cultures from adult woody plants (Cuenca et al., 1999; Corredoira et al., 2015). Maturation and quality of somatic embryos are further limiting factors in conversing embryos into plants.

### Synthetic Seeds

The concept of synthetic seeds was first mentioned by Murashige (1977). The synthetic or artificial seed was defined as an encapsulated single somatic embryo inside a matrix covering. Later, synthetic seeds of alfalfa were successfully produced by encapsulating somatic embryos in alginate hydrogel (Redenbaugh et al., 1984). In early studies, synthetic seeds referred only to the somatic embryos that were used in plant production and were transported to the field. Following this utilization of synthetic seeds, shoot apical tips, axillary buds, and nodal segments have also been employed as appropriative alternatives to somatic embryos (Sarkar and Naik, 1998; Standardi and Piccioni, 1998; Ara et al., 2000; Danso and Ford-Lloyd, 2003; Rai et al., 2008a, 2009). Until now, synthetic seeds have been used in some economically significant woody species including fruit and forest trees (Prewein and Wilhelm, 2003; Malabadi and Staden, 2005; Singh et al., 2007; Rai et al., 2009).

The encapsulation technology provides the somatic embryo with protection from mechanical damage and a supply of nutrients for the growing embryo. Synthetic seeds could, therefore, be easily handled for storage, transport, and sowing, the same as a zygotic seed (Rai et al., 2009). Hydrated and desiccated forms of encapsulation technology were employed in synthetic seeds production (Sharma et al., 2013). Most efforts involve using different coating agents to encapsulate the propagules. These agents include sodium alginate, potassium alginate, sodium pectate, and carrageenan among others, with sodium alginate being the most common (Malabadi and Staden, 2005; Pintos et al., 2008; Gantait et al., 2015). Carbon sources, plant growth regulators, and antimicrobial agents were also added to the hydrogel to facilitate growth and increase the survival rate of the encapsulated propagules (Bapat and Mhatre, 2005; Rai et al., 2008b). Finally, the encapsulated propagules were cultured on media or in the field for plantlet conversion. Desiccated encapsulation was only suitable for some specious whose somatic embryos are tolerant of desiccation. This indicates that the survival competence of somatic embryos is an important factor for storage and conversion under low moisture conditions. After inducing desiccation tolerance, somatic embryos are coated with a protective and nutritive layer to avoid mechanical damage and provide nutrients during the early stages of conversion (Sharma et al., 2013). This desiccated encapsulation method of generating synthetic seeds is not, however, widely used in woody plants because the somatic embryos of some woody plants cannot tolerate desiccation.

Using somatic embryos of woody plants as explants presents a major problem in that the use of encapsulated embryos results in a lower conversion rate when compare with other explants, such as the shoot tip or nodal segment (Tsvetkov et al., 2006; Germanà et al., 2011). Moreover, different woody species also have different conversion rates. For example, in the case of *Quercus robur* synthetic seeds, the conversion rate was only 26%, but for *Citrus nobilis* × *Citrus deliciosa* and *Pinus patula*, the conversion rates were as high as 80% or more (Malabadi and Staden, 2005; Singh et al., 2007). The genotype of the somatic embryo, the encapsulating agent used, and the matrix determined the success of synthetic seed technology in woody plants (Gantait et al., 2015). Evidence also suggests that the conversion rate may decrease with increased storage times and storage temperatures (Singh et al., 2007; Pintos et al., 2008).

### Cryopreservation

Plant somatic embryos can be preserved in liquid nitrogen, keeping them in a physical state at -196°C (Kartha et al., 1988). Cryopreservation is an effective technique for long-term conservation of woody plant somatic embryos (Lambardi et al., 2008). The key requirement of cryopreservation is that the water content of the cells be kept low enough to prevent the formation of ice crystals, ensuring that somatic embryos can easily recover after storage in liquid nitrogen.

In contrast with one-step direct immersion in liquid nitrogen, slow cooling is the common method for cryopreservation of somatic embryogenic cultures in conifer and broad-leaf trees (Klimaszewska et al., 1992; Cyr et al., 1994; Pérez et al., 1997; Ozudogru et al., 2010). However, slow cooling through a gradual temperature decrease, usually at a rate of -1°C/min up down to -40°C, before immersion in liquid nitrogen requires an expensive controlled-rate freezer (Ozudogru and Lambardi, 2016). Slow cooling is, therefore, both expensive and tedious, making it essential that a simple and reliable cryopreservation method be developed for widespread use in somatic embryo cultures of woody plants.

In order to trigger cell vitrification, two strategies were employed during one-step freezing. Chemical dehydration using highly concentrated vitrification solutions, such as dimethyl sulfoxide or sucrose, is one method, and another is physical dehydration by exposing the somatic embryo to sterile air or silica gel (Sisunandar et al., 2010). Sakai et al. (1990), a plant vitrification solution (PVS2) was developed to induce cell vitrification of navel orange (*Citrus sinensis* Osb.) nucellar cells. This work was carried out to develop a simple and reliable protocol for cryopreservation of woody plants, and has led to successful cryopreservation of embryogenic cells from a large number woody plant species including both angiosperms and gymnosperms (Cyr, 1999; Touchell et al., 2002; Martínez et al., 2003; Barra-Jiménez et al., 2015; San José et al., 2015). To rapidly thaw cryopreserved samples, the cryovials are plunged into warm water. Cryoprotectants are removed from the thawed somatic embryogenic cultures through gradual elution, and the cultures are transferred onto fresh regrowth media. This technique could preserve embryogenic cells for an extended period. For instance, in *A. glutinosa* the vitrification protocol developed for the cryopreservation of embryogenic cultures did not affect plant regeneration potential through somatic embryogenesis as assessed using flow cytometry (San José et al., 2015).

In recent years, cryopreservation has been routinely employed for long-term conservation of plant genetic resources in woody plants (Benelli et al., 2013). Several new cryopreservation techniques have been developed. Encapsulation–dehydration and encapsulation–vitrification procedures are based on the technology developed for the production of synthetic seeds (Rai et al., 2009). These techniques have been applied in several woody species including cork oak (*Quercus suber*; Fernandes et al., 2008), citrus (Gonzalez-Arnao et al., 2003), grapevine (*Vitis* spp.; Wang Q. et al., 2004), and cassava (*Manihot esculenta* Crantz; Charoensub et al., 2004).

### Proteomic Analysis of Somatic Embryogenesis in Selected Woody Plants

In the last decade, the development of a number of proteomic methods has facilitated further understanding of somatic embryogenesis in woody plants (Correia et al., 2016a). High resolution two-dimensional gel electrophoresis and mass spectrometry were employed to identify proteins involved in somatic embryo competence and development for several woody plants including *Quercus suber* (Gomez-Garay et al., 2013), grapevine (*Vitis vinifera*; Marsoni et al., 2008; Zhang et al., 2009), sweet orange (*C. sinensis*; Pan et al., 2009), tamarillo (*Cyphomandra betacea*; Correia et al., 2012), avocado (*Persea americana* Mill.; Guzmán-García et al., 2013), larch (*Larix principis-rupprechtii* Mayr; Zhao et al., 2015), cacao (*Theobroma cacao*; Noah et al., 2013; Niemenak et al., 2015), oil palm (*Elaeis guineensis*; de Carvalho Silva et al., 2014), and *Pinus pinaster* (Morel et al., 2014).

Investigation of changes in protein expression focused mainly on three aspects of somatic embryogenesis: protein expression changes during early stages of embryogenesis, differentially expressed proteins in embryogenic and non-embryogenic cells, and differentially expressed proteins in somatic and zygotic embryos. Numerous proteins involved in a variety of somatic embryogenesis cellular processes have been identified in different woody species. These proteins were classified into the following functional categories based on their primary biological process: (1) stress response; (2) storage proteins; (3) cell proliferation and cell wall metabolism; (4) metabolism and energy state; (5) protein synthesis and processing; (6) signal transduction (Tchorbadjieva, 2016). During the somatic embryo induction stage, embryogenic and non-embryogenic calli have been employed to identify differentially expressed proteins. Stress-related proteins were found to play a critical role in the acquisition of somatic embryogenesis. In grapevine iron-deficiency-responsive proteins, acidic ascorbate peroxidase and isoflavone reductase-like proteins were predominantly expressed in embryogenic callus (Zhang et al., 2009). In tamarillo, metabolism-related proteins, heat-shock, and ribosomal proteins were expressed exclusively or predominantly in embryogenic callus (Correia et al., 2012). In larch, several proteins involved in metabolism and development process, such as ADP-ribosylation factor GTPase-activating proteins, triosephosphate isomerase, and proliferating cell nuclear antigen were significantly upregulated in embryogenic callus (Zhao et al., 2015). These results suggest that embryogenic cells are better able to remove reactive oxygen species and to adapt to a stress state.

Several studies have compared somatic and zygotic embryos, and a differential expression of stress- and storage-related proteins has been identified. In the date palm (*Phoenix dactylifera* L.), protein identified in zygotic embryogenesis and somatic embryogenesis showed that most proteins in the somatic embryo belong to the glycolysis pathway. The zygotic embryo was characterized by the presence of carbohydrate biosynthesis, storage proteins and stress related proteins. Up-regulation of stress related proteins was also observed in somatic embryos (Sghaier-Hammami et al., 2009), as were cell proliferation and cytoskeleton remodeling proteins associated with the primary growth of somatic embryos. In the proliferation stage of somatic embryo from *Quercus suber*, proteins involved in cell division were up-regulated. Reactive oxygen species also play a role in proliferation during this stage, while other proteins like cinnamyl alcohol dehydrogenase and pathogenesis-related protein 5 are implicated in embryonic competence. In the cotyledonary stage, reactive oxygen species detoxification enzymes are activated and reserve products are accumulated. In the mature stage, ethylene accumulation regulates embryo development (Gomez-Garay et al., 2013).

## Key Regulatory Genes in Somatic Embryogenesis

Although somatic embryogenesis is already widely applied, the molecular mechanism initiating and controlling this process requires further study. Compared with model plant *Arabidopsis thaliana*, it is difficult to understand the molecular mechanism regulating somatic embryogenesis in woody plants, especially in forest trees, because of their large size, long life cycle, and large genome size (Merkle and Dean, 2000). Moreover, a lack of effective defective mutants and genome sequences hinder the application of a number of powerful genetic approaches (Trontin et al., 2016). The identification of key genes in *Arabidopsis* will help us to understand the regulatory network of somatic embryogenesis in woody plants.

### Positive Regulator Genes

In *Arabidopsis*, there are four main types of transcription factors involved in somatic embryogenesis. First, *LEAFY COTYLEDON* (*LEC*) gene, including *AtLEC1* and *AtLEC2* were identified based on their loss-of-function mutant phenotypes in embryo identity and seed maturation processes (Lotan et al., 1998; Harada, 2001; Stone et al., 2001). *AtLEC1* encodes the HEME-ACTIVATED PROTEINS (HAP3) subunit of the CCAAT box-binding transcription factor, and ectopic *AtLEC1* expression is sufficient to induce somatic embryogenesis from vegetative cells (Lotan et al., 1998; Gaj et al., 2005). AtLEC1 was found to promoting the auxin pathway by up-regulating the auxin biosynthesis genes *AtYUCCA10* (Junker et al., 2012). Recent studies suggest that *AtLEC1* acts as a central regulator of cell fate determination and integrates diverse signaling pathways like hormone and light signaling pathways with both somatic and zygotic embryogenesis (Braybrook and Harada, 2008; Junker et al., 2012; Radoeva and Weijers, 2014; Huang et al., 2015a,b). *AtLEC2*, *FUSCA3* (*AtFUS3*), and *ABSCISIC ACID INSENSITIVE3* (*AtABI3*) encode B3-domain proteins (Swaminathan et al., 2008). Ectopic expression of *AtLEC2* or *AtFUS3* is result in the exhibition of embryonic traits in adult tissues (Luerßen et al., 1998; Stone et al., 2001). A key set of downstream genes differentially regulated by AtLEC2 and AtFUS3 are associated with developmental control of hormone accumulation. AtLEC2 functions as a regulator of embryogenesis by repressing expression of *Gibberellin 3-beta-dioxygenase 2* (*AtGA3ox2*) and promoting the auxin pathway by up-regulating the auxin biosynthesis genes *AtYUCCA2* and *AtYUCCA4*, and auxin signaling gene *INDOLE-3-ACETIC ACID INDUCIBLE 30* (*AtIAA30*; Curaba et al., 2004; Braybrook et al., 2006; Stone et al., 2008). *AtFUS3* negatively regulates gibberellic acid (GA) accumulation by repressing GA biosynthesis genes *AtGA3ox1* and *AtGA3ox2* (Curaba et al., 2004; Gazzarrini et al., 2004). *AtABI3*, which functions in the regulation of ABA-responsive genes during seed development, was positively regulated by *AtLEC1*, *AtLEC2*, and *AGAMOUS-like 15* (*AtAGL15*) (To et al., 2006; Zheng et al., 2009).

The second group of transcription factors involved in somatic embryogenesis includes *AtAGL15*, which encodes a MADS-box transcription factor expressed in the embryo, ectopic overexpression of which enhanced somatic embryo initiation from the shoot apical meristem (Heck et al., 1995; Harding et al., 2003). AtLEC2 may directly induce expression of *AtAGL15* (Braybrook et al., 2006). *AtAGL15* reduced GA levels by promoting *AtGA2ox6* expression that inactivates GA (Wang H. et al., 2004), and upregulates *IAA30*, which is involved in the promotion of somatic embryo development (Zheng et al., 2009).

The third group includes, *BABY BOOM* (*AtBBM*) that encodes an APETALA2 (AP2) domain transcription factor that is preferentially expressed in developing embryos and seeds. Ectopic expression of *BBM* induces the formation of somatic embryos from leaf and cotyledon margins (Boutilier et al., 2002). *EMBRYOMAKER* (*AtEMK*) is another member of the AP2 gene family and is expressed in developing and mature embryos. *AtEMK* is able to induce the formation of embryo-like structures from cotyledons when ectopically overexpressed (Tsuwamoto et al., 2010).

The final group includes genes from the *WUSCHEL-related homeobox* (*WOX*) gene family that have also been shown to play roles in promoting somatic embryogenesis by activating *AtLEC* genes (Zuo et al., 2002; Wang et al., 2009). These results suggest that *AtWUS* has specialized functions in various developmental processes in plants, such as embryogenic patterning and stem cell maintenance (Gallois et al., 2004; Su et al., 2009).

In addition to these four groups, other positive regulators also play a role in somatic embryogenesis. R2R3-type MYB transcription factors *PLANT GROWTH ACTIVATOR 37* (*PGA37*)/ *AtMYB118* and *AtMYB115* positively regulate *AtLEC1* expression and induce the formation of somatic embryos (Wang et al., 2009). The *SOMATIC EMBYOGENESIS RECEPTOR KINASE1* (*AtSERK1*) gene encodes a leucine-rich repeat transmembrane receptor-like kinase that might be involved in a somatic embryogenesis signaling pathway by forming a protein complex with AtAGL15 (Karlova et al., 2006). *AtSERK1* overexpression increased the efficiency of somatic cell initiation (Hecht et al., 2001).

### Negative Regulator Genes

Several suppressors of somatic embryogenesis have also been isolated, including the B3 VP1/ABI3-LIKE (AtVAL) proteins. The *val1 val2* double-mutant forms embryonic calli in roots and shoots, suggesting that *AtVAL1* and *AtVAL2* play a role in the repression of embryonic developmental programs by repressing *AtLEC* genes (Suzuki et al., 2007). AtVAL proteins may suppress the initiation of embryonic genes via recruiting Polycomb repressive complex 1 (PRC1)-mediate histone H2A ubiquitination (H2Aub) and then maintain repression by PRC2-mediated histone H3 lysine 27 trimethylation (H3K27me3; Yang et al., 2013).

*PICKLE* (*AtPKL*), which encodes a chromodomain/helicase/DNA binding domain (CHD3) chromatin-remodeling factor, is a component of the GA signaling pathway and acts as a negative regulator of embryonic identity by repressing expression of *AtLEC* genes (Ogas et al., 1999; Dean Rider et al., 2003). Mutations in *AtPKL* resulted in different explants of seedlings generating embryogenic calli in the absence of exogenous hormones (Henderson et al., 2004). *AtPKL* gene epigenetically regulates somatic embryogenesis by regulating H3K27me3 levels (Zhang et al., 2012). A recent study revealed that AtPKL acts as a critical node that integrates light, brassinosteroid, and GA signals to epigenetically regulate hypocotyl growth (Zhang D. et al., 2014). This work provides a reference framework for studying the molecular mechanisms of somatic embryogenesis in the future and the role light integration and hormone signaling-mediated histone modification play in this process.

Likewise, other epigenetic regulators, AtPRC1 and AtPRC2 also play critical roles in the transition from the embryonic to the post-embryonic stage by suppressing the expression of *AtLEC* genes (Chen et al., 2010; Bouyer et al., 2011). AtPRC2 mutants spontaneously produce ectopic callus and somatic embryos in tissue culture (Chanvivattana et al., 2004). A recent result shows that AtPRC2 prevents dedifferentiation of mature somatic cells in *Arabidopsis* roots and indicates that fully differentiated cells can also dedifferentiate and produce somatic embryos once AtPRC2 epigenetic repression is removed (Ikeuchi et al., 2015).

### Somatic Embryogenesis Associated Genes in Woody Plants

In order to use molecular methods to improve the process of somatic embryogenesis in woody plants, the effectiveness of the molecular mechanism behind somatic embryogenesis, based on model organisms such as *Arabidopsis*, must be further examined in other species. Therefore, some key genes involved in somatic embryogenesis in woody plants are addressed with emphasis on their possible biological function or as gene markers.

In European larch (*Larix decidua* Mill), several homologous genes of *Arabidopsis BBM*, *LEC1*, *WOX2*, and *SERK* were identified in somatic embryos. Overexpression of *LdLEC1* in *Arabidopsis* influences germination and cotyledon formation, as well as producing a similar phenotype to overexpression of *AtLEC1*, which led to the formation of an embryo-like structure (Lotan et al., 1998). *LdLEC1* and *LdWOX2* are mainly expressed during early embryogenesis, whereas *LdBBM* and *LdSERK* had increased expression later in development (Rupps et al., 2016). Along with *BBM*, *LEC1-like* (*L1L*), *LEC2* and *SERK* were characterized in *T. cacao* (de Oliveira Santos et al., 2005; Alemanno et al., 2008; Zhang Y. et al., 2014; Florez et al., 2015). *TcSERK* was highly expressed in initial induced embryogenic callus (de Oliveira Santos et al., 2005). *TcL1L* transcripts were mainly accumulated in young and immature zygotic or somatic embryos. Ectopic expression of *TcL1L* could partially rescue the *Arabidopsis lec1* mutant phenotype (Alemanno et al., 2008). *TcBBM* expression was observed throughout embryo development. Overexpression of *TcBBM* in *Arabidopsis* and cacao led to phenotypes associated with somatic embryogenesis but in the absence of exogenous hormones (Florez et al., 2015). Likewise, *TcLEC2* was highly expressed in dedifferentiated cells competent for somatic embryogenesis. The overexpression of *TcLEC2* in cacao explants greatly increased the frequency of regeneration of stably transformed somatic embryos (Zhang Y. et al., 2014). Overexpression of the *C. sinensis CsL1L* gene led to similar results (Zhu et al., 2014).

Epigenetic regulation was also important during somatic embryogenesis in woody plants. A recent study on somatic embryogenesis in *Coffea canephora* proposed crosstalk between DNA methylation and histone modifications during the earliest embryogenic stages. Cc*LEC1* and Cc*BBM1* are epigenetically regulated by H3K27me3 (Nic-Can et al., 2013). During somatic embryogenesis in *Quercus suber*, QsPKL and QsVAL1 may be necessary for the correct development of somatic embryos (Pérez et al., 2015). The identification of somatic embryogenesis genes in a range of species that were homologous to those in *Arabidopsis* indicates that the molecular mechanism controlling somatic embryogenesis is conserved between *Arabidopsis* and woody plants.

## Conclusion and Perspectives

**FIGURE 1:**
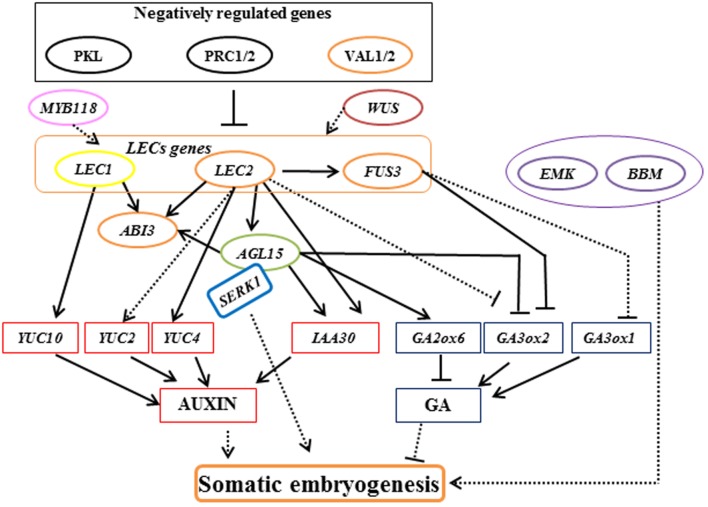
**Model of the regulatory interactions controlling somatic embryogenesis in *Arabidopsis***. Arrows with a solid line indicate direct transcriptional regulation by molecular evidence. Arrows with a dotted line indicate transcriptional regulation that mechanisms are not clear. Orange ellipse represents gene encodes B3-domain proteins. Green ellipse represents gene encodes MADS-box transcription factor. Purple ellipse represents gene encodes AP2 domain transcription factors.

Somatic embryogenesis has the potential to produce plants through *in vitro* propagation and has now become a routine protocol for many trees (Jain et al., 2000; Nawrot-Chorabik, 2012; Germanà and Lambardi, 2016). However, the application of somatic embryogenesis in a wide range of woody plants is limited by genotypic influences, poor germination of somatic embryos, and limited numbers of explants (Isah, 2016). Although progress in characterizing the underlying molecular mechanisms of somatic embryogenesis has been made in *Arabidopsis* and carrot (As shown in **Figure 1**; Zimmerman, 1993; Chugh and Khurana, 2002; von Arnold et al., 2002; Yang and Zhang, 2010; Radoeva and Weijers, 2014; Xu and Huang, 2014; Ikeuchi et al., 2015), studies of woody plants mainly rely on physiological aspects, such as selection of culture media, explant, hormones, and stress substances. Immature and mature zygotic embryos present the most frequently applied source of embryogenic cells that have been used in most of the established protocols of woody plants (Correia et al., 2016b; Isah, 2016).

In the process of somatic embryogenesis, the most critical step is that the somatic cells acquire embryogenic competence. There needs to be a deviation from the normal developmental fate of the cell, which could be considered cell fate transitions of somatic cells under epigenetic regulation (Xu and Huang, 2014). The process of inducing callus from somatic cells treated with 2,4-D and ABA was considered to trigger cell fate transition under stress conditions (Karami and Saidi, 2010; Ikeuchi et al., 2015). However, little is known about the molecular mechanisms of cell fate transition in plants. Further research on this mechanism may help solve the difficulties in transforming somatic cells to embryogenic cells in somatic embryogenesis in woody plants. This requires an understanding of both the biochemical and molecular mechanisms of somatic embryogenesis.

**FIGURE 2:**
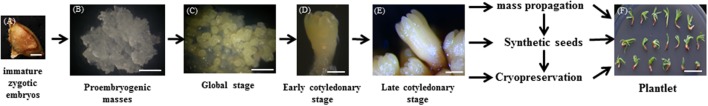
**Schematic overview of Somatic embryogenesis in larch (*Larix* spp.) and applications of somatic embryo**. **(A,B)** proembryogenic masses initiated from immature zygotic embryos. **(C–E)** The microscopic phenotype of somatic embryos at different development stage. **(F)** Plantlet of larch. Scale bars **(A–C)** are 1 mm, **(D,E)** are 0.2 mm, and **(F)** is 1 cm.

The process of somatic embryogenesis of larch and the application of somatic embryos is shown in **Figure 2**. This shows that focus is needed to increase the rate of PEM establishment, and the germination rate of somatic embryos for commercialization. Without this, the somatic embryogenesis technology will have a very limited impact (Isah, 2016). Automation of synthetic seed production is the final goal of commercial seed industries. The combination of a number of factors including advanced tissue culture technology, bioreactors, somatic embryo encapsulation, and development of appropriate synthetic seed coating material requires further study to meet the goal of producing millions of synthetic seeds in a short time and cutting the cost of seed production (Ara et al., 2000; Bapat and Mhatre, 2005; Sharma et al., 2013; Gantait et al., 2015).

With the publication of genome sequences of important woody species, including conifers, broad-leaf trees and fruit trees, more and more important regulatory genes for somatic embryogenesis will be isolated. (Velasco et al., 2010; Nystedt et al., 2013; Verde et al., 2013; Xu et al., 2013; Myburg et al., 2014; Zimin et al., 2014; Plomion et al., 2016). The genomic data complemented by proteomics and transcriptomics will help to comprehend the molecular mechanisms underlying somatic embryogenesis and will allow for the development of more effective *in vitro* regeneration protocols for woody species. In the future, together with outlining the molecular mechanism of somatic embryogenesis in model plants, it is possible to improve the somatic embryogenesis in woody plants using molecular means, independent of plant growth regulators. On the one hand, somatic embryogenesis associated genes could serve as biomarkers for somatic embryogenesis in woody plant tissue to further determine embryogenic initiating competence, particularly for markers that are differentially expressed between embryogenic and non-embryogenic tissue. On the other hand, ectopic expression of somatic embryogenesis associated genes would enhance somatic cell embryogenic potential by transient or constitutive expression. The technology would benefit the propagation of elite trees with low regeneration potential as well as the production of transgenic plants.

## Author Contributions

Z-HS and YG wrote the manuscript. S-GL and X-FF prepared the figure.

## Conflict of Interest Statement

The authors declare that the research was conducted in the absence of any commercial or financial relationships that could be construed as a potential conflict of interest.
